# Crystal structure of tris­(*trans*-1,2-di­amino­cyclo­hexane-κ^2^ *N*,*N*′)cobalt(III) trichloride monohydrate

**DOI:** 10.1107/S2056989015023683

**Published:** 2016-01-01

**Authors:** Megan K. Gallagher, Allen G. Oliver, A. Graham Lappin

**Affiliations:** a Department of Chemistry & Biochemistry, University of Notre Dame, Notre Dame, IN 46556-5670, USA

**Keywords:** crystal structure, cobalt(III), 1,2-di­amino­cyclo­hexa­ne, coordination complex, hydrogen-bonding patterns

## Abstract

The synthesis of the title hydrated complex salt, [Co(C_6_H_14_N_2_)_3_]Cl_3_·H_2_O, from racemic *trans*-1,2-di­amino­cyclo­hexane and [CoCl(NH_3_)_5_]Cl_2_ and its structural characterization are presented in this paper. The product was synthesized in the inter­est of understanding the hydrogen-bonding patterns of coordination complexes. Previous characterizations of the product in the *I*-42*d* space group have not yielded coordinates; thus, this paper provides the first coordinates for this complex in this space group. The octahedrally coordinated cation adopts twofold rotation symmetry, with outer-sphere chloride counter-ions and solvent water mol­ecules forming a hydrogen-bonded network with amine H atoms.

## Abstract 2

The first structure with coordinates of tris­(*trans*-1,2-di­amino­cyclo­hexa­ne)cobalt(III) chloride monohydrate in the space group *I* 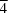
2*d* is reported.

## Chemical context   

We are inter­ested in the hydrogen-bonding patterns of various coordination complexes, especially those that incorporate optically active ligands, where the role of hydrogen bonding in the chiral discrimination between coordination complexes is important. As part of our studies, we prepared the title complex by the reaction of racemic *trans*-(*R*,*R*,*S*,*S*)-1,2-di­amino­cyclo­hexane with [Co(NH_3_)_5_Cl]^2+^ in aqueous solution at 323 K. The resulting complex is a racemic mixture and does not exhibit optical activity. Isolation of optically active forms is being undertaken.
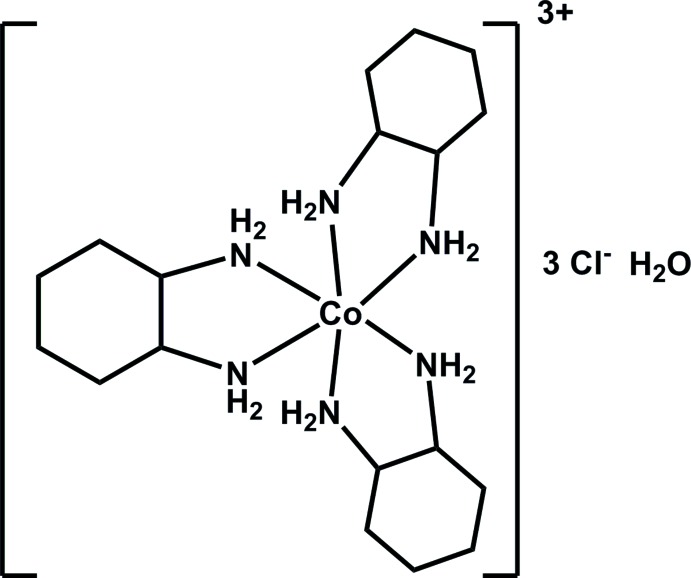


This complex was first reported in 1937 (Jaeger & Bijkerk, 1937 ▸) and by optical crystallography and X-ray diffraction, the space group was determined to be *P*6_1_ and/or *P*6_5_. There have been several, successive studies on this compound, and all are reported in a variety of space groups and configurations of the ligand (*P*6_1(5)_: Jaeger & Bijkerk, 1937 ▸; [*lel_3_*] Marumo *et al.*, 1970 ▸; [*lel_2_ob*] Sato & Saito, 1977 ▸; *C*2: [*ob_3_*] Kobayashi *et al.*, 1972 ▸; *R*32: [*ob_3_*] Kobayashi *et al.*, 1983 ▸; *I* 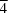
2d, Andersen *et al.*, 1973 ▸). Note: the 1983 Kobyashi article is a correction of the space group reported for the 1972 paper. The Andersen structure was a unit-cell determination and heavy-atom coordinate prediction based on powder diffraction data. No coordinates are available for that structure. Herein, we report the structural characterization of tris­(*trans*-1,2 di­amino­cyclo­hexa­ne)cobalt(III) chloride monohydrate in *I* 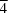
2*d*.

## Structural commentary   

**Figure 1:**
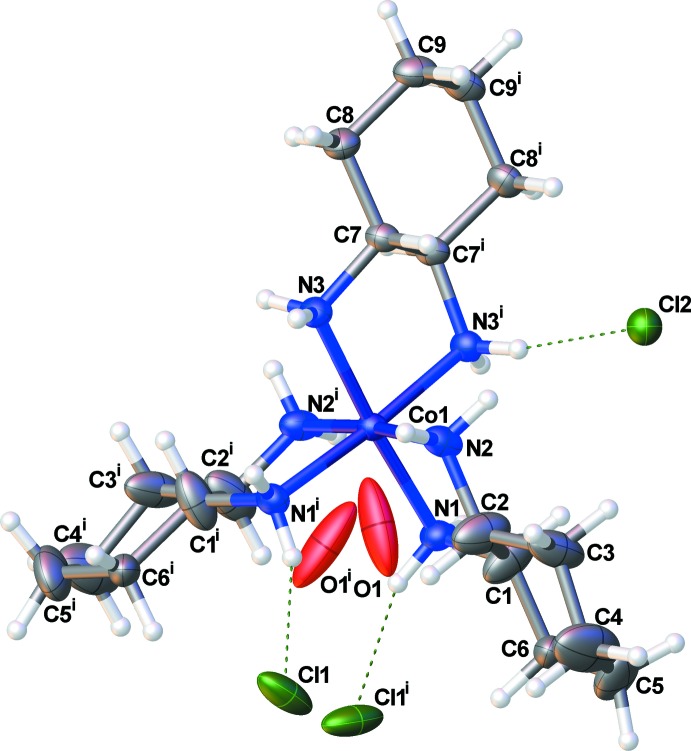
Labeling scheme for (I). Atomic displacement parameters are depicted at the 50% probability level. [Symmetry code: (i) *x*, −*y* + 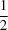, −*z* + 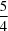.]

The cation crystallizes on a twofold rotation axis at [*x*, 0.25, 0.625], thus, only half of the cation is represented in the asymmetric unit (Fig. 1 ▸). One chloride is located on the twofold axis at [0.75, *y*, 0.875] and the remaining independent chloride anion occupies a general position within the lattice. The water mol­ecule of crystallization is also in a general position, but was modeled as a partial occupancy species (*vide infra*). The 1,2-di­amino­cyclo­hexane ligands adopt a *lel_3_*, Δ (λ,λ,λ) configuration with the (*R*,*R*)-ligand in the featured example. The cobalt atom adopts an octa­hedral coordination environment with only small distortions from an ideal geometry (Table 1 ▸). The 1,2-di­amino­cyclo­hexane ligands are unexceptional.

## Comparison with previously reported structures   

An inspection of the structure and comparison with the Marumo *lel*_3_ complex gives an r.m.s. fit of 0.0706 for the cobalt and nitro­gen atoms (Marumo *et al.*, 1970 ▸; Macrae *et al.*, 2006 ▸). The predominant difference between the Marumo structure and that reported here is the mol­ecular symmetry. The Marumo structure adopts *C*_3_ symmetry, with only one unique ligand. The structure herein adopts *C*_2_ symmetry with one complete and one half ligand in the asymmetric unit.

Perhaps the most surprising change when compared with the Andersen structure is the contraction in cell parameters and overall cell-volume reduction. The cell parameters reported by Andersen are *a* = 19.208, *c* = 13.908 Å, *V* = 5131.3 Å^3^ (Andersen *et al.*, 1973 ▸). Our study has *a* = 18.786, *c* = 13.857 Å and *V* = 4830.3 Å^3^. This change represents a 4.6% reduction in cell volume, with *a* and *b* contracting in a concerted fashion by nearly 0.5 Å. Typically one might expect a contraction of around 0.1 to 0.2 Å upon cooling, similar to that observed for the change in *c*. This observation led us to undertake variable temperature studies to determine if this was actually the case. Data on a crystal of the title compound were recorded at 120 K, 250 K and 293 K. Cell parameters and refinement statistics are given in Table 2 ▸. It should be noted that the redetermination of the unit cell at room temperature with a single crystal sample yielded a unit cell that is approximately 100 Å^3^ smaller in volume than that calculated originally from powder diffraction data.

## Supra­molecular features   

**Figure 2:**
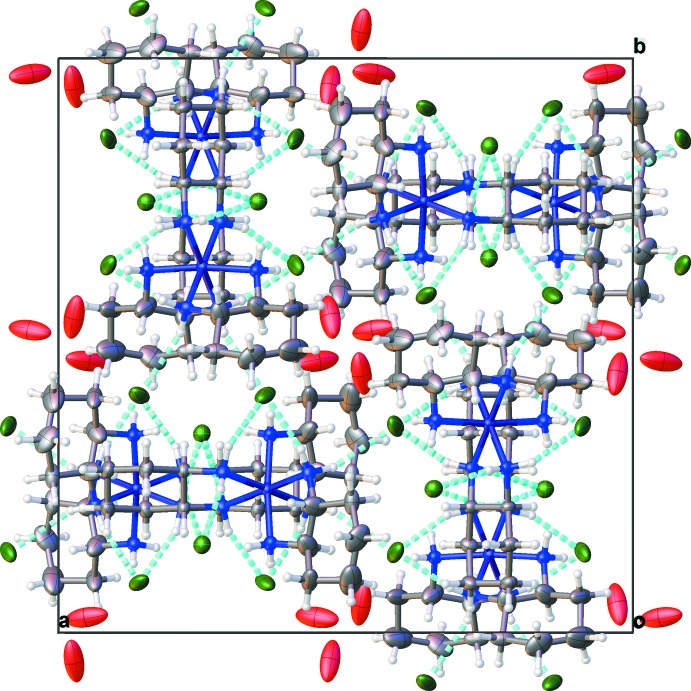
Packing diagram of (I), viewed along the *c* axis. Hydrogen bonds are shown as dashed lines.

The complex forms a hydrogen-bonded network with the amino nitro­gen atoms on the cation serving as donors to nearby chlorine atoms and the water mol­ecule (Fig. 2 ▸, Table 3 ▸). Although the water hydrogen atoms could not be located, there are contacts to nearby chlorine atoms from the oxygen atom at reasonable hydrogen-bond contact distances (Table 3 ▸). Close inspection of the Fourier difference map reveals several locations for potential hydrogen-atom sites on the water oxygen. However, none of these sites refines suitably when modeled as a hydrogen atom. Further exacerbating this situation is the disorder apparent with this lattice water mol­ecule, because through symmetry there is another water oxygen atom located only 2.11 Å distant. Clearly this is unreasonable and reflects the disorder in this mol­ecule. The water of crystallization and chlorine anions are arranged within discrete pockets within the lattice. Other contacts are simple van der Waals inter­actions.

## Database survey   

This structure was first reported in 1937 (Jaeger & Bijkerk, 1937 ▸) with the space group *P*6_1_ and *P*6_5_ at room temperature. Other reports of the structure with the *P*6_1_ space group were in 1970 (Marumo *et al.*, 1970 ▸) and 1977 (Sato & Saito, 1977 ▸), both at room temperature. The structure was also reported in 1972 (Kobayashi *et al.*, 1972 ▸) with the *C*2 space group and 1983 (Kobayashi *et al.*, 1983 ▸) with the *R*32 space group. The first report of the structure with the *I* 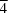
2d space group was in 1973 (Andersen *et al.*, 1973 ▸). This structure is at room temperature and no coordinates were provided by the authors. The structure presented in this paper has the same *I* 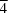
2d space group and provides coordinates for the structure at cryogenic temperatures.

## Synthesis and crystallization   

0.56 g of [Co(NH_3_)_5_Cl]Cl_2_ was dissolved in 200 mL of DI water and allowed to stand overnight. 1.54 g of racemic *trans*-(*R*,*R*,*S*,*S*)1,2-di­amino­cyclo­hexane was added along with a small amount of charcoal. The mixture was stirred and heated at 313–323K for 2 d. The solution was filtered through a SP Sephadex C25 column. Using first 0.01 *M* HCl then 1 *M* HCl, the product was collected from the column. The fractions were placed in evaporation dishes and allowed to dry for three weeks. Orange crystals formed in the evaporation dish and were harvested for analysis.

## Refinement   

Crystal data, data collection and structure refinement details are summarized in Table 4 ▸. All non-hydrogen atoms were refined with anisotropic atomic displacement parameters. Hydrogen atoms were included in geometrically calculated positions with *U*_iso_(H) = 1.2*U*_eq_(C/N). C—H distances were fixed at 0.95 Å and N—H distances fixed at 0.91 Å.

The water of crystallization was determined to be partially occupied by inspection of the displacement parameters during refinement. The occupancy was set to 50% in the final model which yielded reasonable displacement parameters. Hydrogen atoms could not be located or reliably modeled on the water molecule, but have been included in the chemical formula for completeness.

## Supplementary Material

Crystal structure: contains datablock(s) I. DOI: 10.1107/S2056989015023683/lh5797sup1.cif

Structure factors: contains datablock(s) I. DOI: 10.1107/S2056989015023683/lh5797Isup2.hkl

CCDC reference: 1441534

Additional supporting information:  crystallographic information; 3D view; checkCIF report

## Figures and Tables

**Table 1:** Selected geometric parameters (Å, °)

Co1—N1	1.959 (4)	Co1—N2	1.974 (4)
Co1—N1^i^	1.959 (4)	Co1—N3^i^	1.980 (4)
Co1—N2^i^	1.974 (4)	Co1—N3	1.980 (4)
			
N1—Co1—N1^i^	92.2 (3)	N2^i^—Co1—N3^i^	92.64 (19)
N1—Co1—N2^i^	90.61 (19)	N2—Co1—N3^i^	91.62 (19)
N1^i^—Co1—N2^i^	85.4 (2)	N1—Co1—N3	175.8 (2)
N1—Co1—N2	85.4 (2)	N1^i^—Co1—N3	91.58 (17)
N1^i^—Co1—N2	90.61 (19)	N2^i^—Co1—N3	91.62 (19)
N2^i^—Co1—N2	174.2 (3)	N2—Co1—N3	92.64 (19)
N1—Co1—N3^i^	91.58 (17)	N3^i^—Co1—N3	84.7 (3)
N1^i^—Co1—N3^i^	175.8 (2)		

Symmetry code: (i) 
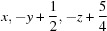
.

**Table 2:** Comparison of 120, 250, and 293 K data sets

	120 K	250 K	293 K
*a*, *c* (Å)	18.960 (3), 13.642 (2)	19.039 (9), 13.651 (7)	19.210 (10), 13.567 (8)
Vol (Å^3^)	4903.8	4948.6	5007.1
% Vol change (w.r.t. 293 K)	2.1	1.2	0.0
*R*[*F* ^2^ > 2σ(*F* ^2^)], *wR*(*F* ^2^), *S*	0.0525, 0.1401, 1.028	0.0386, 0.1078, 1.029	0.0586, 0.1617, 1.017

Data recorded on a second crystal selected from same batch. Crystal showed signs of degradation at higher temperatures, presumably due to solvent loss. Inspection of the crystal after 293 K data set showed fracturing within the crystal.

**Table 3:** Hydrogen-bond geometry (Å, °)

*D*—H⋯*A*	*D*—H	H⋯*A*	*D*⋯*A*	*D*—H⋯*A*
N1—H1*A*⋯Cl1^ii^	0.91	2.46	3.270 (5)	148
N1—H1*B*⋯Cl1	0.91	2.33	3.222 (5)	167
N2—H2*A*⋯Cl2^iii^	0.91	2.57	3.433 (5)	159
N2—H2*B*⋯O1	0.91	2.35	3.019 (14)	130
N3—H3*A*⋯Cl2	0.91	2.46	3.352 (5)	167
N3—H3*B*⋯Cl1^i^	0.91	2.36	3.223 (5)	158
O1⋯Cl1^i^			3.296 (18)	
O1⋯Cl1^iv^			3.393 (15)	
O1⋯Cl1^v^			3.287 (12)	
C2—H2⋯Cl1^iv^	1.00	2.78	3.772 (10)	173
C3—H3*C*⋯O1	0.99	2.37	3.039 (15)	124
C8—H8*A*⋯Cl1^vi^	0.99	2.86	3.780 (6)	156
C8—H8*B*⋯Cl2	0.99	2.94	3.762 (6)	141

Symmetry codes: (i) 
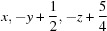
; (ii) 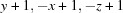; (iii) 
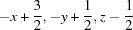
; (iv) 
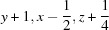
; (v) 
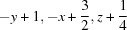
; (vi) 
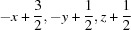
.

**Table 4:** Experimental details

Crystal data	
Chemical formula	[Co(C_6_H_14_N_2_)_3_]Cl_3_·H_2_O
*M*_r_	525.87
Crystal system, space group	Tetragonal, *I* 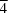 2*d*
Temperature (K)	120
*a*, *c* (Å)	18.7857 (14), 13.8572 (12)
*V* (Å^3^)	4890.2 (9)
*Z*	8
Radiation type	Mo *K*α
μ (mm^−1^)	1.05
Crystal size (mm)	0.22 × 0.06 × 0.05

Data collection	
Diffractometer	Bruker APEXII
Absorption correction	Numerical (*SADABS*; Krause *et al.*, 2015 ▸)
*T*_min_, *T*_max_	0.809, 0.926
No. of measured, independent and observed [*I* > 2σ(*I*)] reflections	43332, 2707, 2416
*R*_int_	0.068
(sin θ/λ)_max_ (Å^−1^)	0.642

Refinement	
*R*[*F* ^2^ > 2σ(*F* ^2^)], *wR*(*F* ^2^), *S*	0.047, 0.130, 1.06
No. of reflections	2707
No. of parameters	137
H-atom treatment	H-atom parameters constrained
Δρ_max_, Δρ_min_ (e Å^−3^)	1.14, −0.62
Absolute structure	Flack *x* determined using 1004 quotients [(*I* ^+^)−(*I* ^−^)]/[(*I* ^+^)+(*I* ^−^)] (Parsons *et al.*, 2013 ▸).
Absolute structure parameter	0.003 (7)

Computer programs: *APEX2* and *SAINT* (Bruker, 2015 ▸), *SHELXT* (Sheldrick, 2015*a* ▸), *SHELXL2014* (Sheldrick, 2015*b* ▸), *OLEX2* (Dolomanov *et al.*, 2009 ▸) and *publCIF* (Westrip, 2010 ▸).

## supplementary crystallographic information

### Crystal data

**Table:**

[Co(C_6_H_14_N_2_)_3_]Cl_3_·H_2_O	*D*_x_ = 1.429 Mg m^−^^3^
*M**_r_* = 525.87	Mo *K*α radiation, λ = 0.71073 Å
Tetragonal, *I*42*d*	Cell parameters from 9897 reflections
*a* = 18.7857 (14) Å	θ = 2.8–24.8°
*c* = 13.8572 (12) Å	µ = 1.05 mm^−^^1^
*V* = 4890.2 (9) Å^3^	*T* = 120 K
*Z* = 8	Rod, orange
*F*(000) = 2240	0.22 × 0.06 × 0.05 mm

### Data collection

**Table:**

Bruker APEXII diffractometer	2707 independent reflections
Radiation source: fine-focus sealed tube	2416 reflections with *I* > 2σ(*I*)
Bruker TRIUMPH curved-graphite monochromator	*R*_int_ = 0.068
Detector resolution: 8.33 pixels mm^-1^	θ_max_ = 27.2°, θ_min_ = 1.8°
combination of ω and φ–scans	*h* = −24→24
Absorption correction: numerical (*SADABS*; Krause *et al.*, 2015)	*k* = −24→24
*T*_min_ = 0.809, *T*_max_ = 0.926	*l* = −17→17
43332 measured reflections	

### Refinement

**Table:**

Refinement on *F*^2^	Secondary atom site location: difference Fourier map
Least-squares matrix: full	Hydrogen site location: inferred from neighbouring sites
*R*[*F*^2^ > 2σ(*F*^2^)] = 0.047	H-atom parameters constrained
*wR*(*F*^2^) = 0.130	*w* = 1/[σ^2^(*F*_o_^2^) + (0.0844*P*)^2^ + 6.4309*P*] where *P* = (*F*_o_^2^ + 2*F*_c_^2^)/3
*S* = 1.06	(Δ/σ)_max_ = 0.010
2707 reflections	Δρ_max_ = 1.14 e Å^−^^3^
137 parameters	Δρ_min_ = −0.62 e Å^−^^3^
0 restraints	Absolute structure: Flack *x* determined using 1004 quotients [(*I*^+^)-(*I*^-^)]/[(*I*^+^)+(*I*^-^)] (Parsons *et al.*, 2013).
Primary atom site location: structure-invariant direct methods	Absolute structure parameter: 0.003 (7)

### Special details

**Table:**

Geometry. All e.s.d.'s (except the e.s.d. in the dihedral angle between two l.s. planes) are estimated using the full covariance matrix. The cell e.s.d.'s are taken into account individually in the estimation of e.s.d.'s in distances, angles and torsion angles; correlations between e.s.d.'s in cell parameters are only used when they are defined by crystal symmetry. An approximate (isotropic) treatment of cell e.s.d.'s is used for estimating e.s.d.'s involving l.s. planes.

### Fractional atomic coordinates and isotropic or equivalent isotropic displacement parameters (Å^2^)

**Table:**

	*x*	*y*	*z*	*U*_iso_*/*U*_eq_	Occ. (<1)
Co1	0.86364 (5)	0.2500	0.6250	0.0199 (2)	
Cl1	0.85979 (10)	0.08706 (8)	0.43019 (16)	0.0553 (5)	
Cl2	0.7500	0.15300 (10)	0.8750	0.0324 (4)	
N1	0.9360 (2)	0.2216 (2)	0.5307 (3)	0.0265 (9)	
H1A	0.9779	0.2126	0.5612	0.032*	
H1B	0.9219	0.1813	0.4996	0.032*	
N2	0.8689 (2)	0.3445 (2)	0.5631 (4)	0.0289 (10)	
H2A	0.8301	0.3513	0.5249	0.035*	
H2B	0.8693	0.3791	0.6090	0.035*	
N3	0.7858 (2)	0.2801 (2)	0.7122 (3)	0.0239 (9)	
H3A	0.7819	0.2488	0.7621	0.029*	
H3B	0.7956	0.3238	0.7370	0.029*	
C1	0.9455 (5)	0.2804 (4)	0.4598 (6)	0.057 (2)	
H1	0.9047	0.2750	0.4141	0.068*	
C2	0.9338 (4)	0.3487 (5)	0.5050 (7)	0.063 (2)	
H2	0.9740	0.3562	0.5511	0.075*	
C3	0.9375 (4)	0.4089 (4)	0.4326 (7)	0.060 (2)	
H3C	0.9309	0.4549	0.4662	0.071*	
H3D	0.8986	0.4036	0.3849	0.071*	
C4	1.0086 (5)	0.4087 (6)	0.3809 (9)	0.092 (4)	
H4A	1.0473	0.4181	0.4277	0.110*	
H4B	1.0094	0.4468	0.3316	0.110*	
C5	1.0206 (5)	0.3362 (5)	0.3323 (6)	0.065 (2)	
H5A	0.9874	0.3319	0.2772	0.078*	
H5B	1.0696	0.3351	0.3061	0.078*	
C6	1.0107 (3)	0.2723 (4)	0.3971 (4)	0.0418 (17)	
H6A	1.0058	0.2289	0.3570	0.050*	
H6B	1.0532	0.2665	0.4385	0.050*	
C7	0.7181 (3)	0.2825 (3)	0.6575 (4)	0.0251 (10)	
H7	0.7186	0.3259	0.6157	0.030*	
C8	0.6525 (3)	0.2853 (3)	0.7199 (4)	0.0311 (12)	
H8A	0.6529	0.3296	0.7587	0.037*	
H8B	0.6523	0.2443	0.7648	0.037*	
C9	0.5856 (3)	0.2833 (3)	0.6569 (5)	0.0359 (13)	
H9A	0.5840	0.3263	0.6157	0.043*	
H9B	0.5428	0.2833	0.6985	0.043*	
O1	0.9506 (10)	0.4729 (6)	0.6332 (9)	0.104 (6)	0.5

### Atomic displacement parameters (Å^2^)

**Table:**

	*U*^11^	*U*^22^	*U*^33^	*U*^12^	*U*^13^	*U*^23^
Co1	0.0181 (4)	0.0195 (4)	0.0221 (4)	0.000	0.000	−0.0026 (4)
Cl1	0.0471 (9)	0.0311 (8)	0.0877 (13)	−0.0106 (7)	0.0231 (10)	−0.0252 (8)
Cl2	0.0324 (9)	0.0358 (10)	0.0291 (9)	0.000	0.0006 (8)	0.000
N1	0.023 (2)	0.030 (2)	0.027 (2)	0.0010 (18)	0.0021 (17)	−0.0049 (18)
N2	0.022 (2)	0.024 (2)	0.041 (2)	−0.0001 (18)	−0.003 (2)	0.0034 (18)
N3	0.023 (2)	0.024 (2)	0.025 (2)	0.0015 (18)	0.0006 (17)	−0.0056 (17)
C1	0.071 (5)	0.043 (4)	0.055 (4)	0.003 (4)	0.032 (4)	0.013 (3)
C2	0.050 (4)	0.058 (5)	0.080 (6)	0.014 (4)	0.023 (4)	0.035 (4)
C3	0.030 (3)	0.057 (5)	0.091 (6)	−0.003 (3)	−0.006 (4)	0.046 (4)
C4	0.055 (5)	0.104 (9)	0.117 (9)	−0.008 (5)	0.008 (6)	0.070 (8)
C5	0.068 (5)	0.086 (7)	0.042 (4)	−0.026 (5)	0.017 (4)	0.014 (4)
C6	0.026 (3)	0.074 (5)	0.026 (3)	−0.001 (3)	0.000 (2)	0.009 (3)
C7	0.020 (2)	0.027 (2)	0.029 (2)	0.000 (2)	−0.0004 (19)	−0.002 (2)
C8	0.025 (3)	0.037 (3)	0.031 (3)	0.005 (2)	0.004 (2)	−0.001 (2)
C9	0.025 (3)	0.036 (3)	0.047 (3)	0.005 (2)	0.005 (2)	0.003 (3)
O1	0.206 (18)	0.049 (6)	0.058 (7)	0.026 (9)	−0.076 (10)	−0.016 (6)

### Geometric parameters (Å, º)

**Table:**

Co1—N1	1.959 (4)	C3—C4	1.516 (12)
Co1—N1^i^	1.959 (4)	C3—H3C	0.9900
Co1—N2^i^	1.974 (4)	C3—H3D	0.9900
Co1—N2	1.974 (4)	C4—C5	1.536 (15)
Co1—N3^i^	1.980 (4)	C4—H4A	0.9900
Co1—N3	1.980 (4)	C4—H4B	0.9900
N1—C1	1.489 (8)	C5—C6	1.510 (11)
N1—H1A	0.9100	C5—H5A	0.9900
N1—H1B	0.9100	C5—H5B	0.9900
N2—C2	1.462 (9)	C6—H6A	0.9900
N2—H2A	0.9100	C6—H6B	0.9900
N2—H2B	0.9100	C7—C8	1.506 (7)
N3—C7	1.482 (6)	C7—C7^i^	1.516 (10)
N3—H3A	0.9100	C7—H7	1.0000
N3—H3B	0.9100	C8—C9	1.531 (8)
C1—C2	1.445 (12)	C8—H8A	0.9900
C1—C6	1.508 (9)	C8—H8B	0.9900
C1—H1	1.0000	C9—C9^i^	1.532 (13)
C2—C3	1.513 (9)	C9—H9A	0.9900
C2—H2	1.0000	C9—H9B	0.9900
			
N1—Co1—N1^i^	92.2 (3)	C3—C2—H2	106.6
N1—Co1—N2^i^	90.61 (19)	C2—C3—C4	110.6 (6)
N1^i^—Co1—N2^i^	85.4 (2)	C2—C3—H3C	109.5
N1—Co1—N2	85.4 (2)	C4—C3—H3C	109.5
N1^i^—Co1—N2	90.61 (19)	C2—C3—H3D	109.5
N2^i^—Co1—N2	174.2 (3)	C4—C3—H3D	109.5
N1—Co1—N3^i^	91.58 (17)	H3C—C3—H3D	108.1
N1^i^—Co1—N3^i^	175.8 (2)	C3—C4—C5	109.9 (8)
N2^i^—Co1—N3^i^	92.64 (19)	C3—C4—H4A	109.7
N2—Co1—N3^i^	91.62 (19)	C5—C4—H4A	109.7
N1—Co1—N3	175.8 (2)	C3—C4—H4B	109.7
N1^i^—Co1—N3	91.58 (17)	C5—C4—H4B	109.7
N2^i^—Co1—N3	91.62 (19)	H4A—C4—H4B	108.2
N2—Co1—N3	92.64 (19)	C6—C5—C4	115.2 (7)
N3^i^—Co1—N3	84.7 (3)	C6—C5—H5A	108.5
C1—N1—Co1	108.8 (4)	C4—C5—H5A	108.5
C1—N1—H1A	109.9	C6—C5—H5B	108.5
Co1—N1—H1A	109.9	C4—C5—H5B	108.5
C1—N1—H1B	109.9	H5A—C5—H5B	107.5
Co1—N1—H1B	109.9	C1—C6—C5	111.2 (7)
H1A—N1—H1B	108.3	C1—C6—H6A	109.4
C2—N2—Co1	109.3 (4)	C5—C6—H6A	109.4
C2—N2—H2A	109.8	C1—C6—H6B	109.4
Co1—N2—H2A	109.8	C5—C6—H6B	109.4
C2—N2—H2B	109.8	H6A—C6—H6B	108.0
Co1—N2—H2B	109.8	N3—C7—C8	114.1 (4)
H2A—N2—H2B	108.3	N3—C7—C7^i^	106.2 (3)
C7—N3—Co1	109.3 (3)	C8—C7—C7^i^	111.7 (4)
C7—N3—H3A	109.8	N3—C7—H7	108.2
Co1—N3—H3A	109.8	C8—C7—H7	108.2
C7—N3—H3B	109.8	C7^i^—C7—H7	108.2
Co1—N3—H3B	109.8	C7—C8—C9	110.0 (4)
H3A—N3—H3B	108.3	C7—C8—H8A	109.7
C2—C1—N1	110.7 (6)	C9—C8—H8A	109.7
C2—C1—C6	117.6 (7)	C7—C8—H8B	109.7
N1—C1—C6	113.7 (6)	C9—C8—H8B	109.7
C2—C1—H1	104.4	H8A—C8—H8B	108.2
N1—C1—H1	104.4	C8—C9—C9^i^	110.4 (4)
C6—C1—H1	104.4	C8—C9—H9A	109.6
C1—C2—N2	108.5 (7)	C9^i^—C9—H9A	109.6
C1—C2—C3	111.6 (7)	C8—C9—H9B	109.6
N2—C2—C3	116.4 (6)	C9^i^—C9—H9B	109.6
C1—C2—H2	106.6	H9A—C9—H9B	108.1
N2—C2—H2	106.6		
			
Co1—N1—C1—C2	32.0 (8)	C2—C3—C4—C5	56.6 (11)
Co1—N1—C1—C6	167.1 (5)	C3—C4—C5—C6	−51.3 (11)
N1—C1—C2—N2	−45.9 (10)	C2—C1—C6—C5	−43.7 (10)
C6—C1—C2—N2	−179.1 (6)	N1—C1—C6—C5	−175.6 (6)
N1—C1—C2—C3	−175.5 (6)	C4—C5—C6—C1	43.1 (10)
C6—C1—C2—C3	51.3 (11)	Co1—N3—C7—C8	164.5 (4)
Co1—N2—C2—C1	37.8 (8)	Co1—N3—C7—C7^i^	41.0 (6)
Co1—N2—C2—C3	164.7 (7)	N3—C7—C8—C9	−176.7 (5)
C1—C2—C3—C4	−57.2 (11)	C7^i^—C7—C8—C9	−56.2 (7)
N2—C2—C3—C4	177.5 (9)	C7—C8—C9—C9^i^	57.0 (7)

Symmetry code: (i) *x*, −*y*+1/2, −*z*+5/4.

### Hydrogen-bond geometry (Å, º)

**Table:**

*D*—H···*A*	*D*—H	H···*A*	*D*···*A*	*D*—H···*A*
N1—H1*A*···Cl1^ii^	0.91	2.46	3.270 (5)	148
N1—H1*B*···Cl1	0.91	2.33	3.222 (5)	167
N2—H2*A*···Cl2^iii^	0.91	2.57	3.433 (5)	159
N2—H2*B*···O1	0.91	2.35	3.019 (14)	130
N3—H3*A*···Cl2	0.91	2.46	3.352 (5)	167
N3—H3*B*···Cl1^i^	0.91	2.36	3.223 (5)	158
O1···Cl1^i^			3.296 (18)	
O1···Cl1^iv^			3.393 (15)	
O1···Cl1^v^			3.287 (12)	
C2—H2···Cl1^iv^	1.00	2.78	3.772 (10)	173
C3—H3*C*···O1	0.99	2.37	3.039 (15)	124
C8—H8*A*···Cl1^vi^	0.99	2.86	3.780 (6)	156
C8—H8*B*···Cl2	0.99	2.94	3.762 (6)	141

Symmetry codes: (i) *x*, −*y*+1/2, −*z*+5/4; (ii) *y*+1, −*x*+1, −*z*+1; (iii) −*x*+3/2, −*y*+1/2, *z*−1/2; (iv) *y*+1, *x*−1/2, *z*+1/4; (v) −*y*+1, −*x*+3/2, *z*+1/4; (vi) −*x*+3/2, −*y*+1/2, *z*+1/2.
